# Autologous peritoneo-fascial patch for portal vein and inferior vena cava reconstruction: a case report with video vignette

**DOI:** 10.1093/jscr/rjaf981

**Published:** 2025-12-12

**Authors:** Rui Ferreira-Santos, Cláudio Branco, Nuno Gonçalves, Nuno Machado, Ana Peixoto Pereira, Mariana Silva Costa, Carlos Veiga, Joaquim Costa Pereira

**Affiliations:** Department of General Surgery, Unidade Local de Saúde de Braga, Sete Fontes - São Victor, 4710-243 Braga, Portugal; Department of General Surgery, Unidade Local de Saúde de Braga, Sete Fontes - São Victor, 4710-243 Braga, Portugal; Department of General Surgery, Unidade Local de Saúde de Braga, Sete Fontes - São Victor, 4710-243 Braga, Portugal; Department of General Surgery, Unidade Local de Saúde de Braga, Sete Fontes - São Victor, 4710-243 Braga, Portugal; Department of General Surgery, Unidade Local de Saúde de Braga, Sete Fontes - São Victor, 4710-243 Braga, Portugal; Department of General Surgery, Unidade Local de Saúde de Braga, Sete Fontes - São Victor, 4710-243 Braga, Portugal; Department of General Surgery, Unidade Local de Saúde de Braga, Sete Fontes - São Victor, 4710-243 Braga, Portugal; Department of General Surgery, Unidade Local de Saúde de Braga, Sete Fontes - São Victor, 4710-243 Braga, Portugal

**Keywords:** hepatobiliary surgery, venous reconstruction, autologous peritoneo-fascial patch, hepatectomy, autologous patch

## Abstract

Venous reconstruction with an autologous peritoneo-fascial patch (APFP) is a valuable technique during hepatobiliary surgery that enables resection with clear margins with readily available autologous tissue. This report details a case of an extended right hepatectomy with segment 1 resection and venous reconstruction using an APFP in a 42-year-old male with an intrahepatic cholangiocarcinoma invading the portal vein (PV) and inferior vena cava (IVC). The surgical approach included resection of the PV and IVC, and type 2 reconstruction with an APFP harvested from the left hypochondrium. The patch was sutured with 4–0 polypropylene, maintaining venous calibre and patency. Figures and an illustrative video of the venous reconstruction are shown. Histopathology confirmed an R0 resection (ypT4N1). This case demonstrates the feasibility of using an APFP for venous reconstruction and highlights its potential for curative resection in advanced hepatobiliary oncologic surgery.

## Introduction

The objectives of oncologic liver resection are to achieve clear margins while preserving adequate function of the remaining liver [1]. Venous reconstruction with autologous peritoneo-fascial patch (APFP) is a feasible and valuable technique enabling resection with clear margins and venous reconstruction with readily available autologous tissue [2]. Here, we present a review of a case of an extended right hepatectomy combined with hepatic segment 1 resection, and venous resection with type 2 reconstruction with an APFP in a patient with intrahepatic cholangiocarcinoma with invasion of the portal vein (PV) and inferior vena cava (IVC).

## Case report

### Case description

**Figure 1:**
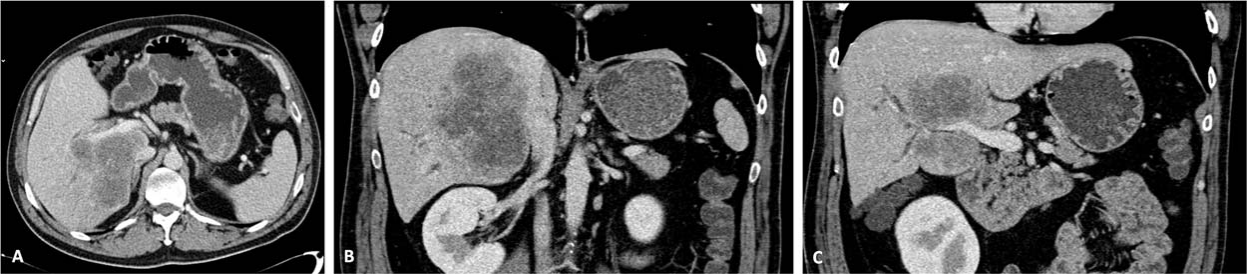
CT scan of the intrahepatic cholangiocarcinoma before chemotherapy. (A) Axial CT scan of the intrahepatic cholangiocarcinoma centred in liver segments 5, 7, and 8, with extension to segment 1 and invasion of the PV and IVC; (B) coronal CT scan view of the vascular relation between the cholangiocarcinoma and the IVC; and (C) vascular invasion of the PV.

A 42-year-old asymptomatic male patient was referred with a suspicious liver mass detected on abdominal ultrasound (US). The US was made after a blood test revealed an isolated elevation of gamma-glutamyl transferase of 789 IU/l. Past and family history were unremarkable. Computed tomography (CT) scan revealed a large tumour measuring 12.6 cm occupying the right liver (Fig. 1 and Supplementary Video S1). Magnetic resonance confirmed a heterogeneous, ring-enhancing hepatic lesion with obliteration of the right hepatic vein (RHV), and invasion of the middle hepatic vein (MHV), PV, and the retrohepatic portion of the IVC. No metastatic lesions were present. Tumour markers and endoscopic exams revealed no alterations.

**Figure 2:**
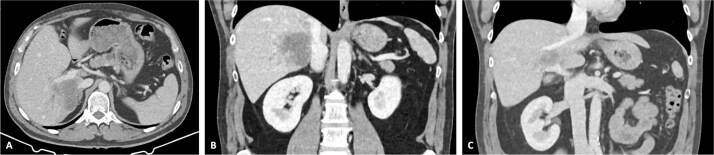
CT scan of the intrahepatic cholangiocarcinoma after chemotherapy. (A) Axial CT scan showing cholangiocarcinoma diameter reduction after chemotherapy and (B) and (C) coronal CT scan view of the vascular relation between the cholangiocarcinoma, IVC, and PV.

The patient was discussed at a multidisciplinary meeting for suspected intrahepatic cholangiocarcinoma. Due to the tumour size and macrovascular invasion, the patient was proposed for neoadjuvant treatment. After a biopsy confirmed the diagnosis, the patient underwent three cycles of gemcitabine and cisplatin, resulting in a reduction of the cholangiocarcinoma from the maximum longitudinal diameter of 12.6 to 7.5 cm (Fig. 2). An extended right hepatectomy with resection and reconstruction of the PV and IVC using an APFP was then proposed.

### Operative technique

The surgery began with an exploratory laparoscopy. Metastatic disease was excluded following inspection of the abdominal cavity. A modified Makuuchi incision was performed. Intraoperative US delineated tumour margins and assessed the vascular relations. The proper hepatic artery divided into three branches: the right hepatic artery (RHA), left hepatic artery, and a middle hepatic artery supplying segment 4. Following cholecystectomy and lymphadenectomy of the hepatic hilum, the RHA was ligated.

**Figure 3:**
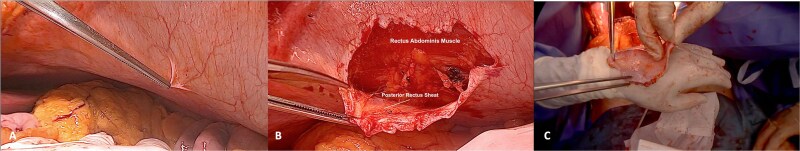
Harvesting of the autologous peritoneo-fascial patch. (A) Selecting of APFP harvesting site—in this case, the APFP was collected from the left hypochondrium; (B) dissection of the APFP between the rectus abdominis muscle and its posterior sheath; and (C) inspection of the harvested APFP.

The APFP, comprising the parietal peritoneum and the posterior rectus sheath, was harvested from the left hypochondrium (Fig. 3). A sufficiently large patch is essential to avoid future constriction of the reconstructed vessel. Preoperative CT showed a tumour with a maximum diameter of 7.5 cm. Assuming invasion of the IVC wall along the entire tumour length, a patch measuring 10 cm in length and 8 cm in width was harvested.

Tangential resection of the PV was then performed, with immediate closure using a 4–0 polypropylene suture. Due to venous thrombosis, a new PV reconstruction was performed using a portion of the sampled peritoneum, with the mesothelial surface facing the lumen, resulting in adequate vessel patency.

Partial right adrenalectomy was necessary for mobilization of the right hepatic lobe. After isolating the suprahepatic and infrahepatic segments of the IVC, parenchymal transection proceeded using ultrasonic energy. The RHV, MHV, right branch of the PV, and right hepatic duct were ligated and transected.

**Figure 4:**
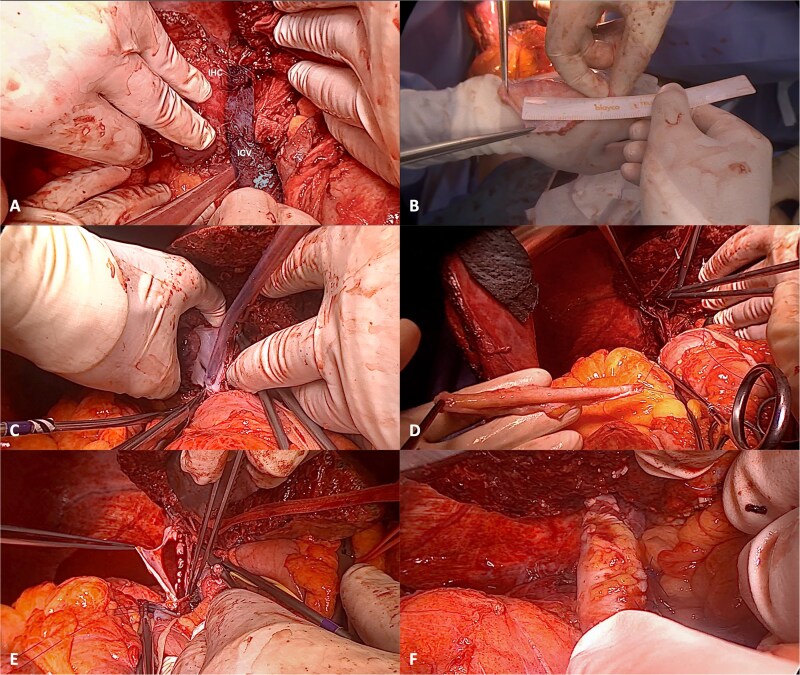
Reconstruction of the IVC with APFP. (A) Intrahepatic cholangiocarcinoma infiltrating the IVC; (B) after assessing the area of IVC resection, the APFP is measured and prepared; (C) following vascular clamping, tangential resection of the IVC anterolateral wall is performed; (D) traction sutures are placed at the borders of the APFP and the corners of the IVC; (E) the APFP patch is sutured using a running 4–0 polypropylene suture; and (F) the vascular clamp is removed, and the reconstructed IVC is inspected for bleeding and stenosis.

**Figure 5:**
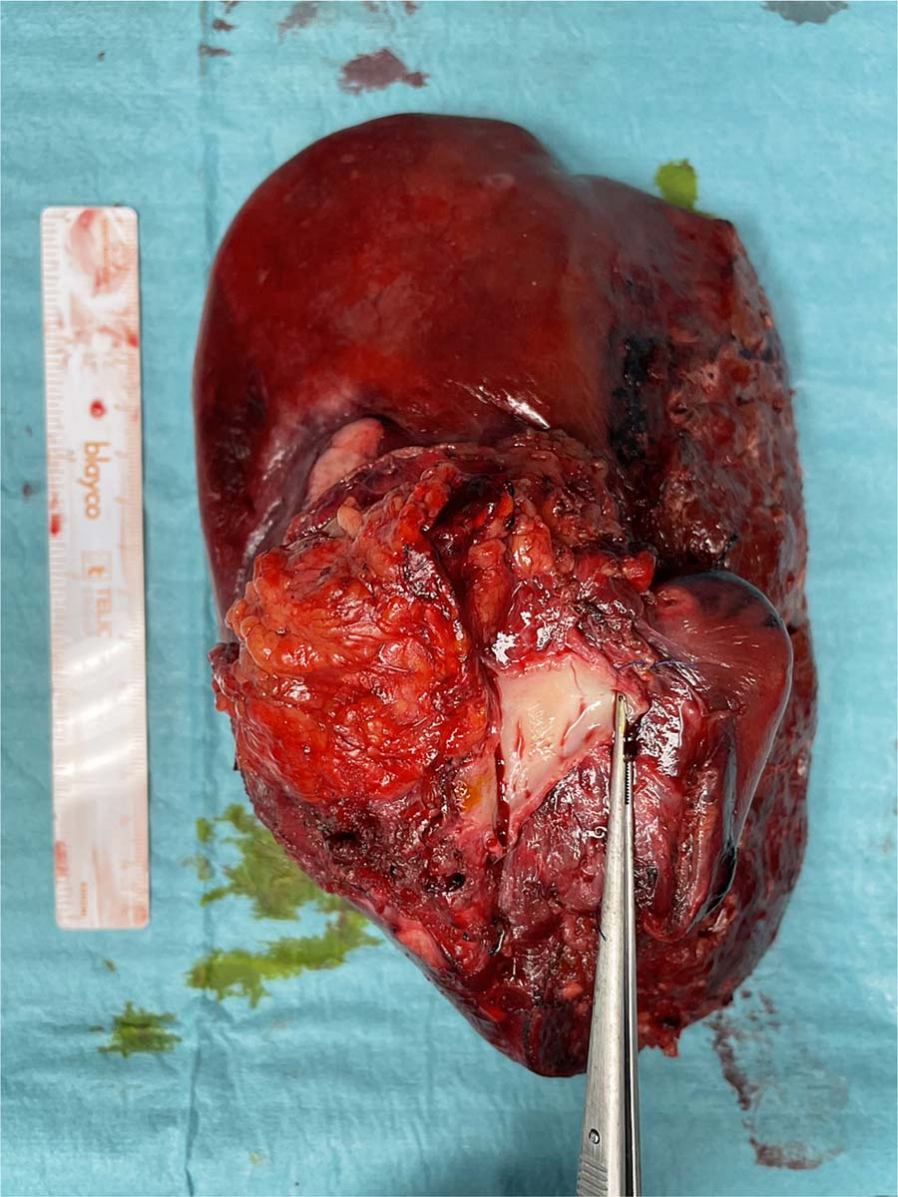
Final specimen. The resected specimen shows intrahepatic cholangiocarcinoma with invasion of the IVC.

Vascular clamps were applied to the IVC. Before resection, we measured the expected vascular defect. An IVC defect measuring 5 cm in length and 2.5 to 3 cm in width was confirmed. The patch was tailored to 6 × 4 cm, allowing a repair larger than the defect to avoid tension and stenosis. We proceeded with resection of the IVC anterolateral wall. Reconstruction using the APFP, with the mesothelial side of the patch facing the lumen, was performed using a running 4–0 polypropylene suture (Fig. 4). It is essential to flush the vein with heparinized saline and avoid suture tension. Total IVC clamping time was 30 min. The video vignette demonstrates the surgical technique. The specimen is shown in Fig. 5.

### Post-operative period

**Figure 6:**
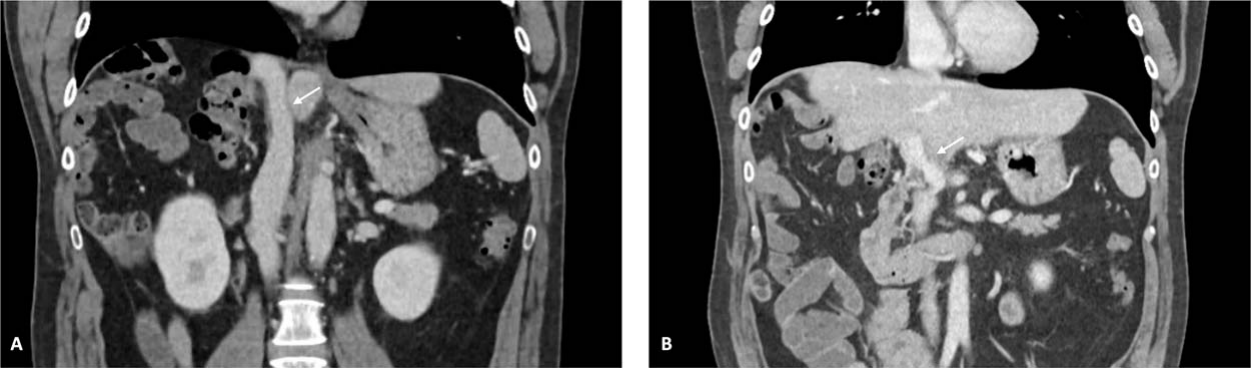
Reconstructed IVC and PV. (A) IVC and (B) PV 1 year after surgery.

The post-operative period was complicated by bleeding from the left epigastric artery at the APFP harvesting site on post-operative day 14, requiring reintervention and vessel ligation. The patient was discharged on post-operative day 31. Pathology confirmed an ypT4G2N1R0 small-duct intrahepatic cholangiocarcinoma. No post-operative thrombosis or clinically significant stenosis were identified. Figure 6 demonstrates the reconstructed vessels 1 year after surgery. After completing 6 weeks of therapeutic low-molecular-weight heparin (LMWH), the patient continued rivaroxaban 20 mg once daily.

Eleven months after surgery, the patient presented with an iliac bone metastasis, with no local recurrence. The patient started antalgic radiotherapy and chemotherapy with gemcitabine, cisplatin, and durvalumab.

## Discussion

Major venous vessel involvement is an important consideration when planning for an R0 resection as radical surgical resection remains the only curative treatment.

Several venous reconstruction techniques are available, employing synthetic, and biological materials [3, 4]. Autologous patches offer several advantages, including immediate availability, low infection risk, low risk of thrombosis, and easy adaptation to vascular defects [2].

Chin first reported the use of peritoneum as a patch for IVC reconstruction in humans in 1999 [3]. Since then, case reports and case series have supported the use of APFP and peritoneal flaps in hepatobiliary surgery and unplanned vascular resections [5–9]. Dokmak published a series of thirty patients who underwent liver or pancreatic resection with venous reconstruction using a peritoneal patch. There were no patch-related complications, all patients achieved R0 resection, and all venous reconstructions were patent after 14 months, except for one tubular graft [5].

No anticoagulation protocols exist for IVC and PV type 2 reconstruction. After PV reconstruction, Radulova-Mauersberger recommends prophylactic LMWH for 4 weeks if vein diameter is ≥1 cm, and half-therapeutic doses if diameter is <1 cm [10]. In our case, intraoperative PV thrombosis prompted initiation of therapeutic LMWH, followed by rivaroxaban. Discontinuation was planned at 1 year, but due to bone metastasis, maintenance of rivaroxaban was decided.

Here, we present an extended right hepatectomy with PV and IVC resection, followed by reconstruction using an APFP. This case highlights intraoperative and post-operative challenges, including thrombosis requiring venous reconstruction and bleeding from the patch harvesting site. By outlining the surgical steps, we aim to promote the adoption of this technique in complex oncologic procedures.

## Supplementary Material

Supplementary_video_1_rjaf981

Video_JSCR_rjaf981
